# A rare cause of recurrent subconjunctival hemorrhage: ocular vicarious menstruation

**DOI:** 10.3205/oc000213

**Published:** 2023-01-30

**Authors:** Ali Riza Cenk Celebi, Elif Ganime Aygun

**Affiliations:** 1 Acibadem University School of Medicine, Department of Ophthalmology, Istanbul, Turkey; 2 Acibadem Atakent Education and Research Hospital Department of Obstetrics and Gynecology, Istanbul, Turkey

**Keywords:** recurrent subconjunctival hemorrhage, ocular vicarious menstruation, extrauterine bleeding, oral contraceptive pills

## Abstract

**Purpose:**

Vicarious menstruation is cyclical bleeding in extra-uterine locations that occurs during menstruation or within 48 h of its onset. We aim to present a 43-year-old female with ocular vicarious menstruation, its treatment, and a review of other published cases of ocular vicarious menstruation.

**Case description:**

A 43-year-old Caucasian female presented with a 15-year history of recurrent monthly unilateral subconjunctival hemorrhage. The episodes were cyclical and coincided with the onset of menses, lasting for approximately 10 to 14 days. Slit-lamp examination of the right eye showed nasally located subconjunctival hemorrhage. Detailed laboratory findings, including parameters for various hematological disorders, were normal. A follow-up examination 2 weeks later showed that the subconjunctival hemorrhage in the right eye was completely resolved. The patient was prescribed the oral contraceptive levonorgestrel/ethinyl estradiol and marked improvement at the recurrences of subconjunctival hemorrhage was noted during subsequent menses.

**Conclusion:**

Ocular vicarious menstruation is among the rarest causes of recurrent subconjunctival hemorrhage. A therapeutic trial of oral contraceptive should be considered in patients that present with ocular vicarious menstruation.

## Introduction

Subconjunctival hemorrhage is characterized by the acute appearance of a flat area of blood under the conjunctiva. Subconjunctival hemorrhage is diagnosed clinically and typically requires no treatment, clearing within a few weeks with good visual prognosis [1]. However, if hemorrhage recurs, an extensive list of differential diagnoses must be considered, as there can be an underlying cause. Recurrent subconjunctival hemorrhage was reported to occur in various circumstances due to forceful coughing, sneezing, lifting, bending, vomiting, standing on head, and taking drugs like aspirin, NSAIDS, coumadin, heparin, and also such in trauma, eye rubbing, systemic diseases like diabetes, hypertension, bleeding and clotting disorders. The major known risk factors for recurrent subconjunctival hemorrhage are trauma and contact lens-induced eye injuries in young patients, whereas hypertension is the most common risk factor in elderly patients [2]. Other causes of recurrent subconjunctival hemorrhage include hematological dyscrasias, use of anticoagulation medications and antiplatelet medications, idiopathic thrombocytopenic purpura, and genetic variation of various blood coagulation factors [3]. 

When recurrent subconjunctival hemorrhage consistently occurs every month in association with menstruation, it is referred to as ‘ocular vicarious menstruation’, which is among the rarest causes of subconjunctival hemorrhage [4]. Vicarious menstruation is a rare condition characterized by cyclical bleeding that occurs in extra-genital organs during menstruation. The most common site of such bleeding is the nasal mucosa, but there have been rare reports of bleeding from other sites, including the eyes [4]. Only 1% of women with vicarious menstruation have ocular involvement [4]. Vicarious menstruation usually occurs during the 4th and 5th decades of life [5]. There have only been a few case reports of vicarious menstruation in ocular structures [3], [6], [7], [8], [9]. Here, we present a female patient with ocular vicarious menstruation (which to the best of our knowledge is the oldest such patient to be reported), its treatment, and a review of other published cases of ocular vicarious menstruation.

## Case description

A 43-year-old Caucasian female presented with a 15-year history of recurrent monthly spontaneous right-sided subconjunctival hemorrhage. In 2004 she underwent surgery of the left ovary due to ovarian cysts and then from 2004 to 2007 was treated with the oral contraceptive levonorgestrel/ethinyl estradiol. She reported that during the time she used the oral contraceptive, the frequency of recurrent subconjunctival hemorrhage decreased to every three months. However, when she stopped the medication in 2007 recurrent monthly subconjunctival hemorrhage resumed. These cyclical episodes occurred in conjunction with the timing of her menstrual cycle and lasted for approximately 10 to 14 days.

**Figure 1:**
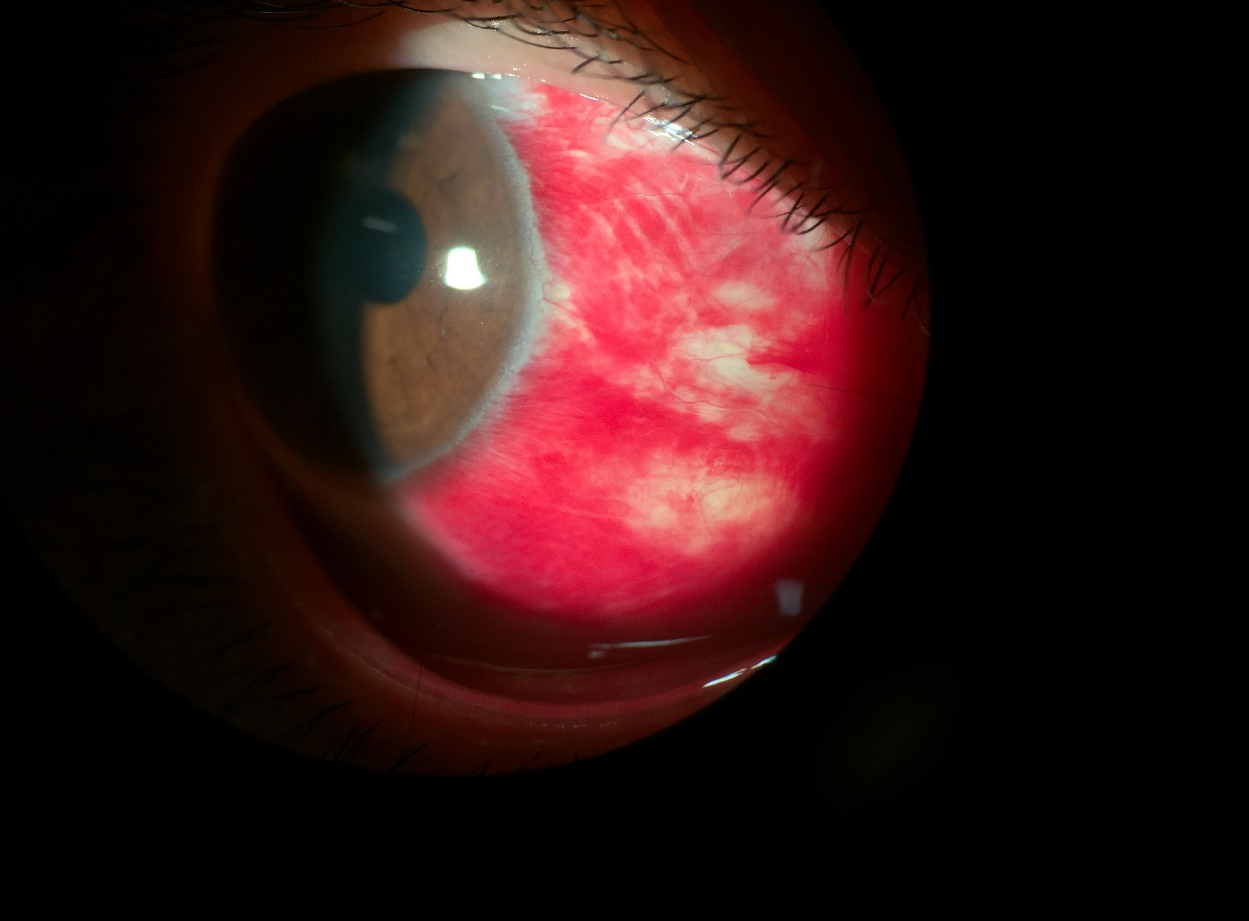
Slit-lamp image of the patient’s right eye at the onset of menstruation shows nasally located subconjunctival hemorrhage.

Family history of ocular and systemic diseases was negative and no precipitating factors, such as uncontrolled bleeding during dental procedures, trauma, easy bruising, easy bleeding, and lack of anticoagulant and antiplatelet medication use, were reported. Ophthalmic examination showed that visual acuity was 20/20 and intraocular pressure was 13 mmHg in both eyes. Extraocular movements and pupillary response to light and accommodation were also normal. Slit-lamp examination of the right eye showed nasal subconjunctival hemorrhage (Figure 1 (Fig. 1)) without chemosis or elevation. Slit-lamp examination of the left eye and dilated funduscopic exam of both eyes were unremarkable.

**Figure 2:**
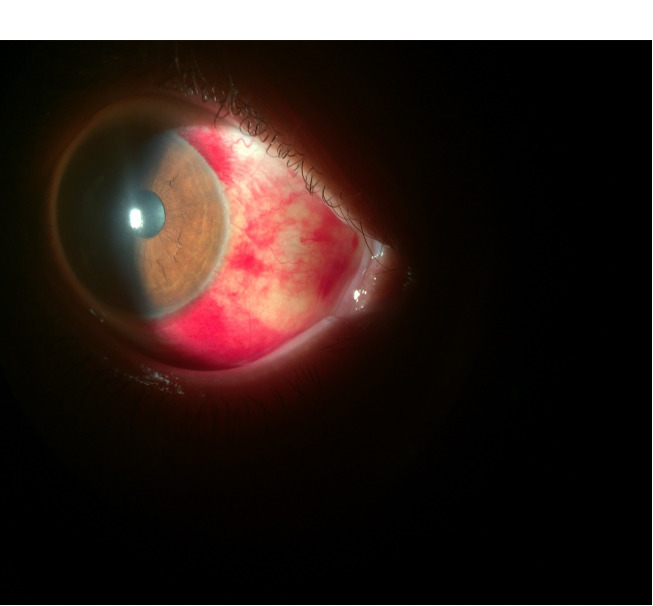
Slit-lamp image of the patient’s right eye 1 week after the onset of menstruation shows that the subconjunctival hemorrhage is beginning to clear.

**Figure 3:**
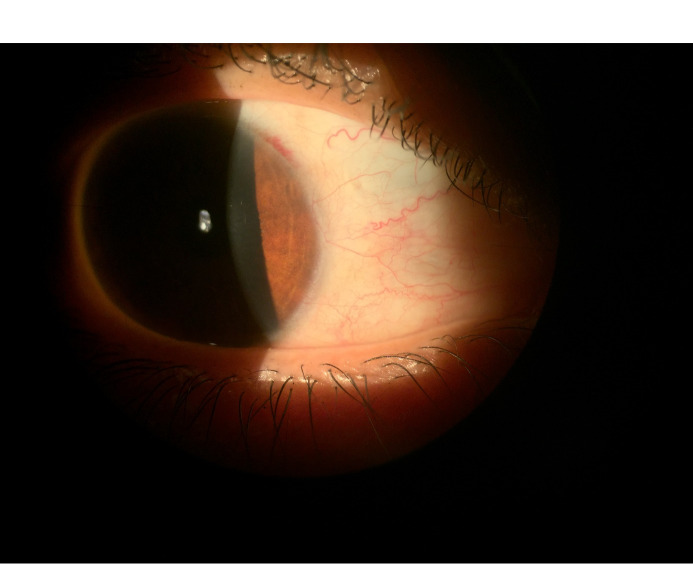
Slit-lamp image of the patient’s right eye 2 weeks after the onset of menstruation shows total resolution of subconjunctival hemorrhage.

Laboratory findings, including protein C, protein S, anti-thrombin III, prothrombin time, partial thromboplastin time, bleeding time, international normalized ratio, and the platelet count, were normal. Orbital MRI during menses was also normal. On follow-up examination after 1 week (at the end of menses), the subconjunctival hemorrhage was beginning to resolve (Figure 2 (Fig. 2)). Two weeks after the initial exam, the subconjunctival hemorrhage in the right eye was completely resolved (Figure 3 (Fig. 3)). The patient was offered conjunctival biopsy, but declined to undergo the procedure. The patient was then treated again with the oral contraceptive levonorgestrel/ethinyl-estradiol and reported marked improvement in the amount of subconjunctival hemorrhage during subsequent menses.

## Discussion

The reported causes of recurrent subconjunctival hemorrhage include amyloidosis, lacrimal tumors, vascular tumors, arteriovenous malformations, lymphangiomas, trauma, history of surgery, use of anticoagulant medications, and systemic bleeding disorders [1]. Ocular vicarious menstruation is a rare condition characterized by cyclic bleeding that occurs in the eye during menstruation. This phenomenon has been described in various ocular structures, including the extraocular muscles [5], conjunctiva [8], retina[7] , and lacrimal apparatus [10]. Although the etiology and pathophysiology of vicarious menstruation is not well understood, it has been hypothesized to be due to the presence of endometrial tissue at extra-uterine locations with hormonally responsive vasculature.

Estrogen and progesterone can cause congestion, hyperemia, and secondary bleeding in extra-uterine tissues via increasing capillary permeability [5]. Sex steroid hormones, such as estrogen, progesterone, and androgen, might be associated with various ocular pathologies, as their mechanisms of action are via their receptors in ocular tissue. Any pathology that can affect the levels of these sex hormones can affect ocular tissues as well [4]. Conjunctival epithelium exhibits cyclic variation during menstruation and menopause [11]. The estrogen level seems to correlate with the epithelium maturation index [12].

Vicarious menstruation is cyclical bleeding in extra-uterine organs that occurs during menstruation or within 48 h of its onset. The presented patient’s cyclic subconjunctival hemorrhage always occurred within 24 h of the onset of menses. When evaluating a patient with vicarious menstruation extensive workup to identify or rule out local lesions, tumors of the lacrimal apparatus, vascular tumors, and systemic bleeding disorders should be performed. Once these common etiological factors for recurrent hemorrhage are ruled out, the treatment goal should be ovulatory suppression via hormonal therapy if it is not clinically contraindicated [8].

The literature includes only a few reports of ocular vicarious menstruation [5], [6], [8], [9], [10], [13]. In all of the reported cases the diagnostic key was the cyclic nature of the clinical signs and symptoms. Abboud et al. [6] reported vicarious menstruation of the conjunctiva in an anemic 17-year-old patient that received treatment only for anemia. Barat et al. [8] reported a 17-year-old patient with ocular vicarious menstruation. Ophthalmic examination showed blood in the fornix, medial cantus, and lacrimal gland area, but the exact anatomical location that was affected was not specified. Laboratory and radiographic tests did not identify any vascular abnormalities, vascular masses, or intraocular lesions. The patient was successfully treated with the combination oral contraceptive mestranol/norethynodrel. Turkcuoglu et al. [10] described cyclical bleeding in the left inferior punctum in a 13-year-old girl. MRI showed hemorrhagic tissue in the nasolacrimal canal, and she was subsequently diagnosed as presumed nasolacrimal endometriosis. Because of the patient’s age and presence of anovulatory cycles, conservative management was chosen.

Gauba et al. [9] reported a 30-year-old woman with an 8-year history of unilateral recurrent subconjunctival hemorrhage that would begin on the first day of the menstrual cycle and resolve 7 to 10 days later. Slit-lamp examination of her right eye showed a fleshy, thickened, discrete subconjunctival mass in the inferior fornix, which was considered a possible conjunctival lymphoma; therefore, a biopsy specimen was obtained and the histological findings were consistent with conjunctival amyloidosis. Light microscopy of the mass did not show ectopic endometrial tissue. Vascular and lymphatic abnormalities were not noted, an inflammatory response was not observed, and oral contraceptive pills did not stop the bleeding. Systemic examination and laboratory findings, including serum amyloid protein, were unremarkable, suggesting that the amyloid deposition was localized to conjunctival tissue. Although surgical debulking of the lesion temporarily stopped the occurrence of hemorrhage, the symptoms recurred 2 years later, as the mass progressively increased to its original size. The most recently reported case of ocular vicarious menstruation was a 31-year-old African-American woman. The patient presented with a 1-year history of recurrent monthly spontaneous right-sided orbital and subconjunctival hemorrhage, which occurred in conjunction with the timing of her menstrual circle and lasted approximately 7 days. Ophthalmological examination showed non-tender periorbital fullness and diffuse subconjunctival hemorrhage in the right eye. Orbital MRI performed during menses showed enlargement of all right extraocular muscles, without lymphovascular lesions or malformations in the orbit or cavernous sinus. She was treated with a combination estrogen/progesterone oral contraceptive pill, which resulted in a quick clinical response with marked improvement in amount of subconjunctival hemorrhage during subsequent menstrual cycles. The researchers concluded that the patient’s recurrent subconjunctival hemorrhage was due to the presence of estrogen and progesterone receptors in the right orbital extra-uterine endometrial tissue and that the oral contraceptive pill she was treated with led to atrophy of these aberrant tissues and consequent clinical improvement [5]. The presented patient is the seventh and the oldest case of ocular vicarious menstruation in the English-language literature and also had a good response to treatment with oral contraception.

In conclusion, in cases of recurrent subconjunctival hemorrhage it is essential to perform complete clinical, serologic, and radiographic investigations, and when warranted histologic investigation, to determine the precise location of and potential etiological factors for subconjunctival hemorrhage. Ocular vicarious menstruation is among the rarest causes of recurrent subconjunctival hemorrhage. A therapeutic trial of oral contraceptive pills should be considered in patients that present with ocular vicarious menstruation.

## Notes

### Competing interests

The authors declare that they have no competing interests.
